# A nuclear-replicating viroid antagonizes infectivity and accumulation of a geminivirus by upregulating methylation-related genes and inducing hypermethylation of viral DNA

**DOI:** 10.1038/srep35101

**Published:** 2016-10-14

**Authors:** Enza Maria Torchetti, Mattia Pegoraro, Beatriz Navarro, Marco Catoni, Francesco Di Serio, Emanuela Noris

**Affiliations:** 1 Institute for Sustainable Plant Protection, National Research Council of Italy, Bari, 70126, Italy; 2 Institute for Sustainable Plant Protection, National Research Council of Italy, Torino, 10135, Italy; 3 The Sainsbury Laboratory, University of Cambridge, Cambridge, CB2 1LR, United Kingdom

## Abstract

DNA methylation and post-transcriptional gene silencing play critical roles in controlling infection of single-stranded (ss) DNA geminiviruses and ssRNA viroids, respectively, but both pathogens can counteract these host defense mechanisms and promote their infectivity. Moreover, a specific role of DNA methylation in viroid-host interactions is not yet confirmed. Here, using an experimental system where two nuclear-replicating agents, the geminivirus tomato yellow leaf curl Sardinia virus (TYLCSV) and potato spindle tuber viroid (PSTVd), co-infect their common host tomato, we observed that PSTVd severely interferes with TYLCSV infectivity and accumulation, most likely as a consequence of strong activation of host DNA methylation pathways. In fact, PSTVd alone or in co-infection with TYLCSV significantly upregulates the expression of key genes governing DNA methylation in plants. Using methylation-sensitive restriction and bisulfite conversion assays, we further showed that PSTVd infection promotes a strong hypermethylation of TYLCSV DNA, thus supporting a mechanistic link with the antagonism of the viroid on the virus in co-infected tomato plants. These results describe the interaction between two nuclear-replicating pathogens and show that they differentially interfere with DNA methylation pathways.

DNA methylation is an important epigenetic mark that regulates gene expression^1^, genetic imprinting^2^, silencing of transposable elements and frequency of homologous recombination^3^. In plants, DNA methylation occurs at cytosines in symmetric (CG and CHG) and asymmetric (CHH, where H can be A, T, or C) contexts, through maintenance and *de novo* processes. D*e novo* DNA methylation is based on a RNA-dependent DNA methylation (RdDM) mechanism involving 24-nt small interfering RNAs (siRNAs) generated by a DICER-like (DCL) ribonuclease, together with other components of the transcriptional gene silencing (TGS) machinery. The role of RNA in *de novo* DNA methylation was disclosed using an experimental system based on viroids^4^. Viroids are infectious agents with a genome consisting of a small (246–401 nt) circular single-stranded (ss)RNA that does not code for proteins but can move systemically and cause severe diseases in plants^5^. Viroids replicate in the nucleus (family *Pospiviroidae*) or the chloroplast (family *Avsunviroidae*) through rolling-circle mechanisms with double-stranded (ds)RNA intermediates^5^. Like viruses, they are triggers and targets of post-transcriptional gene silencing (PTGS) involved in anti-viroid defense and symptom induction^6^, However, a possible role of RdDM in plant-viroid interactions and the interference of viroids with host DNA methylation are almost unexplored^6^. Viroid-derived 24 nt-small RNAs (vd-sRNAs) identified in tissue infected by a member of the family *Pospiviroidae* are loaded onto an Argonaute protein, AGO4^7^, and can direct DNA methylation on homologous transgenic DNA sequences^8^,^9^. This indicates that viroid RNAs are targeted by the host enzymes DCL and AGO involved in RdDM. More recently, a nuclear-replicating viroid has been reported to dynamically modify DNA methylation of ribosomal RNA (rRNA) genes, opening the perspective that viroids may actually interfere with host RdDM pathways^10^,^11^.

In contrast to viroids, RdDM has a well-documented role against geminiviruses (GVs). These are plant pathogens with an ssDNA genome that forms minichromosomes associated with cellular histones and is transcribed and replicated in the nucleus, with dsDNA intermediates^12^. Some evidences indicate that plants methylate GV DNA as an antiviral response^13^: i) *in vitro* methylated GV DNA is not efficiently replicated and transcribed^14^, ii) hypermethylation of GV DNA is linked to host recovery^15^,^16^, iii) *Arabidopsis* mutants impaired in the methylation pathway are more susceptible to GVs^17^, and iv) GVs encode RNA silencing suppressors (RSSs) that interfere with DNA methylation and counteract plant defense^18^,^19^,^20^. GVs also modify methylation of the host genome, mainly in regions involved in defense and stress responses^21^,^22^, possibly through the action of RSSs.

Numerous plant genomes have been sequenced and assembled; in some instances, cytosine methylation patterns have been determined by whole-genome bisulfite sequencing and mapped to a reference genome, resulting in reference methylomes^23^. However, the identification of differentially methylated genes and their correlation with genetic/phenotypic variations remain a challenge. In addition, it is largely unknown how geminiviruses and viroids, two nuclear replicating pathogens both targeted by enzymes involved in DNA methylation, interact with each other and with host DNA methylation. To fill this gap, we established an experimental system with a GV and a nuclear-replicating viroid infecting a common host. We therefore studied the mutual relationships of these pathogens in terms of infectivity, symptoms and accumulation, focusing on the regulation of methylation-related genes during infection. This approach gave us the opportunity to use the GV genome as a molecular sensor to investigate whether a nuclear-replicating viroid interferes with host DNA methylation. More specifically, we used the begomovirus (family *Geminiviridae*) tomato yellow leaf curl Sardinia virus (TYLCSV)^24^, responsible for destructive diseases, and the potato spindle tuber viroid (PSTVd) (family *Pospiviroidae)*^25^, an emerging threat for potato and tomato crops in Europe. As a common host, we selected tomato whose genome has been recently sequenced^26^.

## Results

### PSTVd antagonizes infectivity and accumulation of TYLCSV in tomato plants

To assess the infectivity, symptoms and relative accumulation of TYLCSV and PSTVd in single and mixed infections, four independent experiments were performed. In each case, groups of six tomato seedlings were singly inoculated with PSTVd and TYLCSV and co-inoculated with both pathogens, using 24 plants for each combination, including controls. Based on molecular hybridization, all plants inoculated with only PSTVd and most (23/24) of those inoculated with only TYLCSV were infected at 6 weeks post-inoculation (wpi). In contrast, all plants co-inoculated with TYLCSV and PSTVd developed PSTVd infection, but only half of them (12/24) were also infected by TYLCSV. The remaining plants were examined for a late TYLCSV infection, but resulted all virus-free even at 11 wpi. None of the 24 plants inoculated with the empty pBIN19 developed virus- or viroid-infection. This indicates that PSTVd exerts an antagonistic effect on TYLCSV infectivity when co-inoculated in tomato plants.

**Figure 1:**
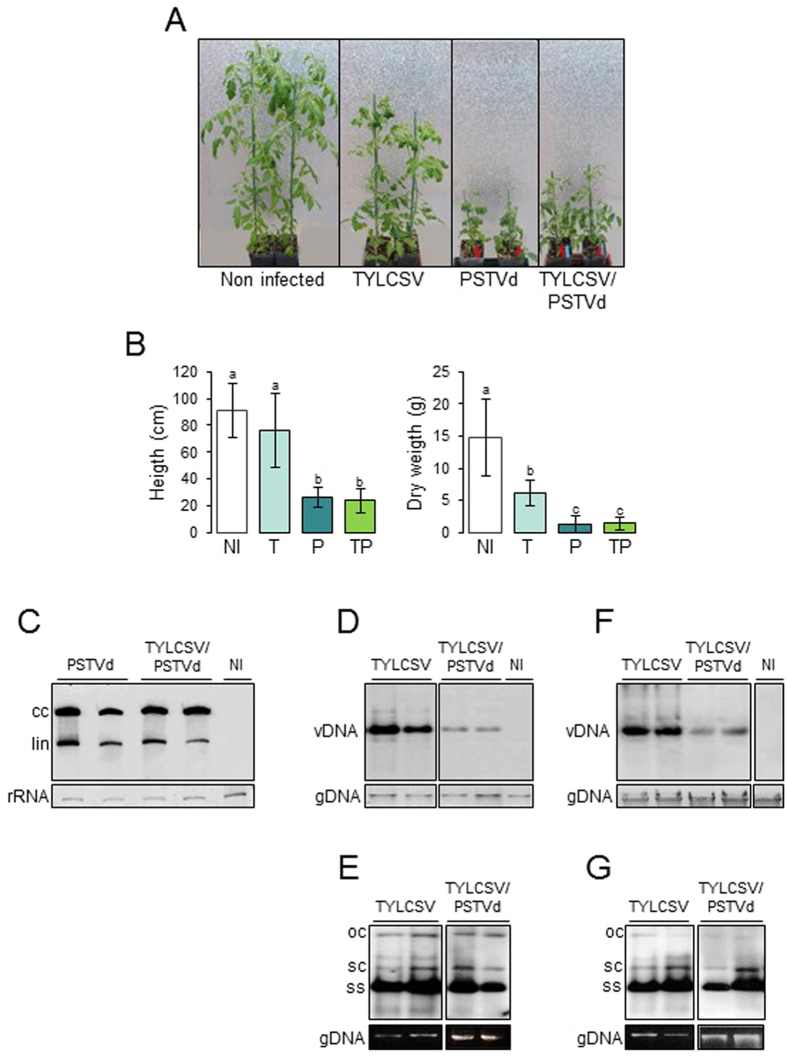
Effect of TYLCSV, PSTVd, or double infection by TYLCSV/PSTVd on tomato plants. (**A**) Symptoms on plants at 9 wpi. Non-infected plants were inoculated with pBIN19, as control. (**B**) Height and dry weight of plants at 11 wpi (12 plants per treatment). NI, non infected plants, T, infected by TYLCSV, P, infected by PSTVd, TP, co-infected by TYLCSV and PSTVd. Bars, standard errors; letters, statistical significance (P ≤ 0.05; Student *t*-test). (**C**) Accumulation of PSTVd in singly- or doubly-infected plants, simultaneously co-inoculated. (**D,E**) Accumulation of TYLCSV in singly-or doubly-infected plants, simultaneously co-inoculated. Agarose gels were run without (**D**) or with (**E**) ethidium bromide, the latter conditions allowing to separate the viral DNA forms. (**F,G**) Accumulation of TYLCSV in plants inoculated first with TYLCSV and superinfected with PSTVd or pBIN19, as control; gels run without (**F**) or with (**G**) ethidium bromide. Samples are always from plants at 6 weeks after TYLCSV inoculation. cc and lin, circular and linear PSTVd forms; rRNA, ribosomal RNA, shown as loading control; vDNA, TYLCSV DNA, including genomic and replicative forms; ss, genomic TYLCSV DNA; oc, sc, open circular and supercoiled TYLCSV dsDNA; gDNA, plant genomic DNA, shown as loading control.

Regarding symptoms, plants infected by TYLCSV alone developed typical leaf deformations, yellowing of the margins, and moderate size reduction, while those infected by PSTVd alone were severely stunted compared to non-infected controls (Fig. 1A). A pronounced size reduction occurred also in plants co-infected by TYLCSV and PSTVd, accompanied by the yellowing and leaf deformations typical of TYLCSV (Fig. 1A). Growth parameters from 12 plants per treatment were evaluated at the end of the experiment, i.e. 11 wpi. Compared to non-infected controls, plants infected by TYLCSV showed a 58.6% dry mass reduction, without significant changes in plant size, while plants infected by PSTVd displayed a 71 and 91% reduction in size and dry weight, respectively (Fig. 1B). Plants harboring TYLCSV and PSTVd showed 73.6% and 90.6% of height and dry weight reduction, respectively, with no synergism.

The strong impact of PSTVd on TYLCSV infectivity led us to analyze the accumulation of both pathogens in plants. Northern-blot hybridization revealed that the amount of circular and linear monomeric RNA molecules of PSTVd was similar in doubly- or singly-infected plants (Fig. 1C). In contrast, the accumulation of TYLCSV DNA was strongly impaired in plants co-infected by TYLCSV and PSTVd compared to those infected by TYLCSV alone (Fig. 1D). Using gel running conditions allowing to discriminate replicative (ds) and genomic (ss) TYLCSV DNA forms, we observed that their ratio was not substantially altered (Fig. 1E). This indicates that PSTVd has a detrimental effect also on the accumulation of both genomic and replicative forms of TYLCSV.

To exclude that this antagonism was a consequence of the inoculation strategy, which relies on the simultaneous release of both pathogens by agrobacterium, we inoculated PSTVd into tomato plants already infected by TYLCSV (inoculated three weeks before). All 15 control plants inoculated with TYLCSV or PSTVd alone became infected and all 15 plants first inoculated with TYLCSV and then with PSTVd developed a mixed infection. However, co-infected plants accumulated a lower amount of TYLCSV DNA at 6 weeks after TYLCSV inoculation, compared to plants singly-infected by TYLCSV (Fig. 1F); again, no substantial difference in the amount of ds and ssDNA were detected (Fig. 1G). Therefore, PSTVd impairs also the progression of an already established TYLCSV infection.

### PSTVd up-regulates the expression of genes involved in DNA methylation pathways

Infection by GVs is linked at various levels to host DNA methylation and viroids have been reported to interfere with transcriptional mechanisms regulated by epigenetic modifications of host DNA^11^. To investigate the mechanism underlying the antagonism of PSTVd towards TYLCSV, we compared the expression of genes involved in DNA methylation and RdDM pathways in infected and non-infected plants. These included Methyltransferase 1 (MET1) and Chromomethylase 3 (CMT3), catalyzing the maintenance of methylation at CG and CHG sites, respectively, and Domains Rearranged Methyltransferase 2 (DRM2), responsible for methylating cytosines in asymmetric contexts through a RdDM mechanism involving 24-nt siRNAs generated by a DCL ribonuclease. Other components of the transcriptional gene silencing (TGS) machinery involved in this process include: i) a conserved S-adenosyl-l-methionine-dependent RNA methyltransferase (Hua enhancer 1, HEN1), which methylates the terminal nucleotide of 24-nt sRNAs, and ii) the core subunits NRPD1 and NRPE1 of the plant specific DNA-dependent RNA polymerases Pol IV and Pol V, respectively, that generate the scaffold RNA templates of the 24-nt siRNAs and recruit the factors required for DNA methylation, respectively^27^. Additional elements involved in RdDM are the RNA-dependent RNA polymerase RDR2, which generates the double-stranded (ds)RNAs targeted by DCLs, and AGO4 that loads the 24-nt siRNAs and specifically guides the RNA-induced silencing complex to DNA target regions^28^. DNA methylation decreases passively during replication if DNA methyltransferases are downregulated, or actively through a base-excision repair pathway mediated by Repressor of Silencing 1 (ROS1)^29^. Other factors participating in DNA methylation are the chromatin remodeler Decreased in DNA Methylation 1 (DDM1), which provides DNA methyltransferases access to histone H1-containing heterochromatin^30^, and the H3K9 histone methyltransferase KRYPTONITE/SUVH4 (KYP), which bridges the histone silencing marks with CMT3-dependent DNA methylation in a positive feedback, while Increase in BONSAI Methylation 1 (IBM1) removes H3K9 methylation^31^,^32^.

Conserved sequences of the functionally annotated genes of *Arabidopsis* available in the TAIR database (https://arabidopsis.org) were identified in the tomato genome database (http://solgenomics.net/), and the homologous genes retrieved. Specific primers were designed within conserved elements to develop quantitative RT-PCR (qRT-PCR) assays (Table 1). The Ubiquitin Conjugating Enzyme (UBC) gene (Table 1) was used as housekeeping (HK) gene, as it showed lack of statistically significant changes in its expression across the experimental conditions (Significativity 0.076; Supplementary Table S1).

**Figure 2:**
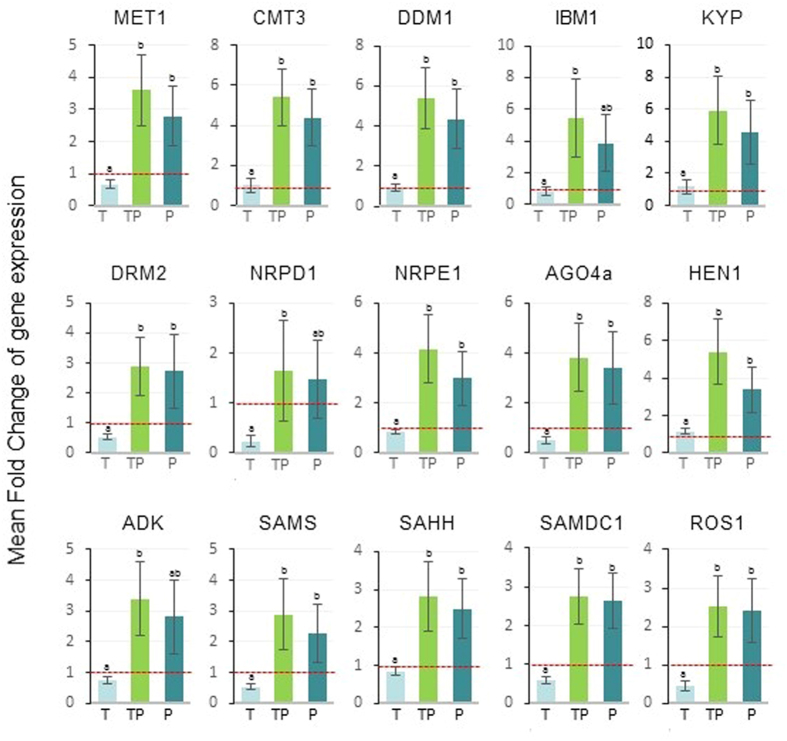
Mean fold change of methylation-related tomato gene expression in plants infected by TYLCSV (T), PSTVd (P) or by TYLCSV/PSTVd (TP), with respect to non-infected control plants. Data are from three infected vs. non-infected plants; the red line set to 1 on the Y axis (FC = 1) means no change in gene expression. Bars, standard errors; letters, statistical significance (P ≤ 0.05; Student *t*-test).

Plants infected by PSTVd and co-infected by PSTVd and TYLCSV exhibited a strong up-regulation of genes involved in the maintenance of DNA methylation (MET1, CMT3, and DDM1) and in histone methylation (KYP and IBM1), compared to non-infected controls and to plants infected by TYLCSV, alone (Fig. 2 and Supplementary Table S2). In the same plants, harboring PSTVd or both PSTVd and TYLCSV, genes involved in *de novo* methylation (DRM2, NRPE1, AGO4, and HEN1), the demethylating enzyme ROS1, as well as genes regulating the availability of methyl groups (ADK, SAHH, and SAMDC1) were also up-regulated (Fig. 2 and Supplementary Table S2). No statistically significant difference in gene expression occurred between plants with PSTVd alone or co-infected by PSTVd and TYLCSV. Conversely, TYLCSV induced a down-regulation of MET1, DRM2, NRPD1, and AGO4a, as well as a decrease in SAMS, SAMDC1, and ROS1, compared to non-infected plants (Fig. 2 and Supplementary Table S2).

Altogether, these data document a differential regulation of tomato genes involved in DNA methylation pathways in response to distinct biological stresses acting separately or concurrently, exploring the molecular interplay between two different nuclear-replicating pathogens and their host.

### PSTVd infection leads to hypermethylation of TYLCSV DNA

**Figure 3:**
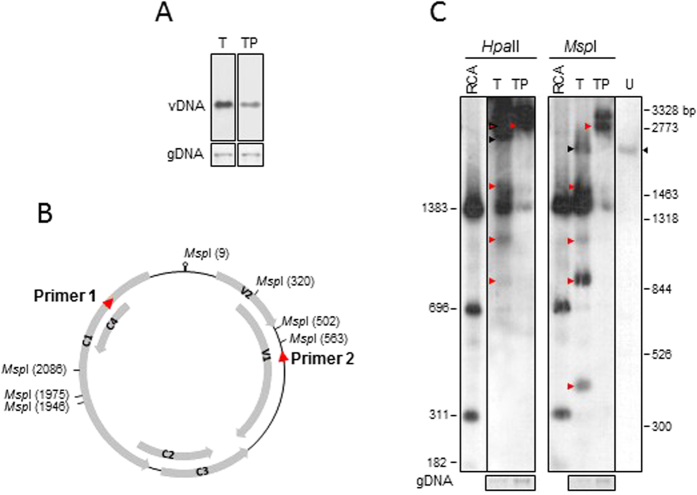
Methylation sensitive restriction enzyme analysis on TYLCSV- or TYLCSV/PSTVd-infected plants. (**A**) TYLCSV DNA accumulation in the plants infected by TYLCSV- or by TYLCSV/PSTVd (T and TP, respectively). (**B**) Position of *Hpa*II and *Msp*I sites in the TYLCSV genome; red arrows, primers to amplify the TYLCSV region for bisulfite analysis (see Fig. 4). (**C**) *Msp*I or *Hpa*II digestion of plant DNA extracts (fragments indicated by red arrows) and of the RCA amplicon (size in bp indicated on the left). U, undigested ss DNA sample with TYLCSV ssDNA (black arrows); TYLCSV size marker (in bp, on the right) are 2773 (*Sst*I digestion of a TYLCSV clone), 3328, 1318, 844 (*Sph*I/*SnaB*I), and 1463, 526, 300 (*Bgl*II/*Sal*I).

Given the up-regulation of host genes involved in DNA methylation, we investigated the presence of hyper-methylated sequence targets using TYLCSV DNA as a molecular sensor. To examine the level of methylation, two strategies - based on methylation-sensitive restriction and bisulfite conversion - were applied to DNA samples from TYLCSV- and TYLCSV/PSTVd-infected plants, the latter having a reduced accumulation of viral DNA (Fig. 3A).

For methylation-sensitive restriction analysis, DNA samples were digested with the isoschizomers *Hpa*II and *Msp*I (Fig. 3B) recognizing the same CCGG sequence. While *Hpa*II cuts only un-methylated DNA, *Msp*I cuts also when the inner cytosine is methylated^33^; none of them cut DNA when both cytosine are methylated. An RCA product generated *in vitro* by TempliPhi DNA polymerase from a TYLCSV-infected plant served as un-methylated DNA control. Following Southern-blot hybridization with a probe specific for the whole TYLCSV genome, we confirmed that the RCA product generated the expected bands of 1383, 696, 311 and 182 bp (Fig. 3C); other bands of 111, 61, and 29 bp were not detected, most likely for their small size. *Hpa*II digestion of samples from plants singly infected by TYLCSV generated the linear TYLCSV genome (2773 bp), plus the 1383-bp fragment observed in the *Hpa*II-digested RCA product; additional bands of about 1600, 1200, and 900 bp are also present, that are not detected in the *Hpa*II-digested RCA product (Fig. 3C). *Hpa*II digestion of extracts from co-infected plants generated mainly the linear TYLCSV genome, plus the 1383-bp fragment. Regarding *Msp*I digestion, singly infected plants produced a pattern similar to that of *Hpa*II, i.e. the 1383, 1600, 1400, and 900 bp fragments, and another of about 400 bp. Co-infected plants generated mostly the linear genomic TYLCSV, plus the 1383- and 1600-bp fragments. Similar results were obtained in two independent experiments. In summary, at least some of the *Hpa*II/*Msp*I sites in the TYLCSV dsDNA appear blocked by methylation at both or external cytosines in singly infected plants, and most of these sites are blocked in the TYLCSV DNA from co-infected plants. These results suggest that the lower accumulation of TYLCSV DNA in doubly- vs. singly-infected plants could be related to an increased level of methylation in viral DNA.

**Figure 4:**
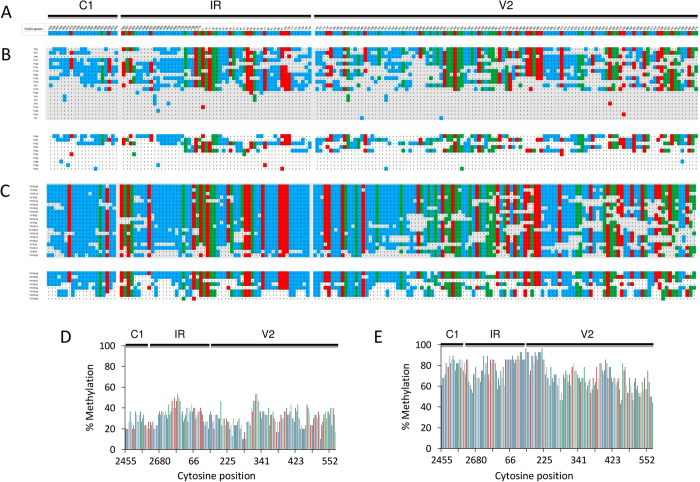
Bisulfite sequencing of TYLCSV DNA. (**A**) TYLCSV fragment (938 nt) including portions of C1 and V1 coding sequences and the intergenic region (IR). (**B,C**) Cytosine methylation in individual clones from TYLCSV- (**B**) or TYLCSV/PSTVd-infected plants (**C**); the two blocks are from two experiments. Methylated cytosines (reported as C) are blue, red and green, if in the CHH, CHG or CG contexts, respectively. Un-methylated cytosines are reported as T. (**D,E**) Percentage of methylated cytosines within the TYLCSV amplicon in TYLCSV- (**D**) or TYLCSV/PSTVd-infected plants (**E**).

Since the methylation-sensitive restriction analysis only detects methylation at specific sites (Fig. 3), we decided to carry out bisulfite sequencing to assess the methylation status of TYLCSV at higher resolution. Bisulfite treatment of DNA deaminates un-methylated cytosines and converts them into uracils, while methylated cytosines remain unchanged^34^. We selected a TYLCSV genome fragment (938  bp) covering the untranscribed intergenic region, the two divergent promoters flanking the origin of replication, and the divergent transcribed regions (Figs 3A and 4A). As expected, more than 99% of cytosines were converted in a TYLCSV-unrelated and un-methylated DNA used as control in each bisulfite reaction (Supplementary Figure S1), indicating the completeness of the reaction in our experimental conditions. For plants infected by TYLCSV alone, out of 30 clones of bisulfite-converted amplicons sequenced from two biological replicates (20 and 10 clones, respectively), 31% of cytosines resulted methylated, on average (Supplementary Figure S2 and Table S3), with some difference between replicates (34.8 and 23.4%). Moreover, a mixture of methylated and un-methylated/hypo-methylated clones (40–50%) was present (Fig. 4B), indicating that TYLCSV molecules with different levels of methylation coexist in the same plant, as previously reported^15^,^19^. In co-infected samples, out of 28 clones from two replicates (20 and 8 clones, respectively), 72.6% of cytosines were methylated (79.1 and 56.5% for each replicate), with all clones highly methylated, except one (Fig. 4C). In all cases, methylated cytosines were uniformly distributed along the viral DNA, independently of the asymmetric (CHH) or symmetric (CG or CHG) context and regardless of whether they mapped into coding or non-coding regions (Fig. 4C,D, and Supplementary Figure S2). The higher cytosine methylation in co-infected plants compared to plants singly infected by TYLCSV is consistent with results from the methylation-sensitive restriction analysis.

Collectively, these data show that hypermethylation of TYLCSV DNA is associated with overexpression of methylation-related genes and with a lower virus accumulation in plants co-infected by PSTVd, suggesting that the antagonism of PSTVd on TYLCSV arises from the stimulation of the host methylation pathways.

## Discussion

In this work, we investigated whether a nuclear replicating viroid interferes dynamically with host DNA methylation pathways in tomato. Due to the lack of suitable markers in the genome of this plant allowing to directly identify changes in the methylation landscape, we used a DNA virus as a methylation sensor. Hence, tomato plants were inoculated with two different pathogens, PSTVd and TYLCSV. Both pathogens replicate in the nucleus and the second one has a DNA genome targeted by host methylation pathways^13^. In addition, they cause severe diseases in tomato and may co-exist in the same crop, as shown by the identification of wild *Solanum* plants naturally infected by tomato yellow leaf curl virus (TYLCV) and PSTVd^35^. This experimental system also allowed to further dissecting the reciprocal interference of the two pathogens in co-infected tomato plants.

Co-infection by viruses and/or viroids may result in synergistic or antagonistic interactions^36^. Synergism was reported in grapevine and citrus plants co-infected by nuclear-replicating viroids and RNA viruses^37^,^38^,^39^. In our case, no synergism occurred in tomato plants co-infected by TYLCSV and PSTVd in terms of symptom severity, measured as plant height or dry-weight (Fig. 1). Rather, PSTVd impaired the infectivity and accumulation of TYLCSV. Interestingly, such antagonism also occurred in plants with an already established TYLCSV infection, indicating that PSTVd usurps factors critical for TYLCSV or stimulates a more efficient defense of the host. Previous experiments of co-infection by the bipartite GV *Abutilon mosaic virus* and PSTVd induced symptom enhancement, but with no changes in viroid or virus titres^40^. Such discrepancy strengthens the notion that co-infections can have different outcomes that are specific for each combination.

In the attempt to elucidate the mechanism underlying the antagonism of PSTVd on TYLCSV and molecularly dissect the plant response to these pathogens, we analyzed the expression of genes required for DNA methylation in plants. The whole set of tomato genes involved in methylation were strongly over-expressed in plants infected by PSTVd and co-infected by PSTVd and TYLCSV, not only compared to healthy plants, but also to plants infected by TYLCSV alone (Fig. 2). Moreover, compared to non-infected controls, TYLCSV down-regulated genes governing *de novo* methylation processes, such as DRM2 and NRPD1, and methyl cycle, such as SAMS and SAMDC1 (Fig. 2). These data confirm that GVs subvert methylation pathways during their infection process^33^ and indicate that plant responses to single and double biotic stresses may differ with no obvious additive effects. The discrepancy concerning the expression of CMT3, reported as downregulated in transgenic *N. benthamiana* following GV infection^41^, can be ascribed to the plant host, the sampling time (a late stage of infection in our case) or the tissue examined, i.e. whole leaf rather than samples enriched in vascular tissue. Interestingly, the strong downregulation of NRPD1 following TYLCSV infection is in line with a recent report where NRPD1, together with NRPE1 (Pol IV and V, respectively) were found relevant for the amplification of methylation and the deposition of H3K9 dimethyl groups on geminivirus chromatin^42^. More importantly, our data support a relationship between viroid infection and host DNA methylation, reinforcing the idea that the viroid activates a plant defense strategy against the geminivirus, based on DNA methylation.

Both plant viruses and viroids interfere directly or indirectly with host gene expression and the transcriptomes of PSTVd- and TYLCSV-infected tomato plants have been analyzed^43^,^44^,^45^,^46^. RNA silencing governs at least part of such changes. Regarding viroids, vd-sRNAs may drive post-transcriptional degradation of host mRNAs and, at least in one case, were shown to impair the expression of a protein involved in pathogenesis^47^. Moreover, 24-nt vd-sRNAs can guide the methylation and transcriptional silencing of a cognate transgene^48^. The clear impact of PSTVd on tomato genes involved in the epigenetic machinery here described allows to include epigenetic pathways among the host biological processes modified by viroids.

Because the antagonism of PSTVd against TYLCSV and the strong induction of methylation-related genes in co-infected plants could be connected, we investigated the level of methylation of the TYLCSV genome in plants under the different infection conditions. The restriction pattern of TYLCSV DNA obtained with methylation-sensitive enzymes and the bisulfite analysis of a region covering about one third of the virus genome showed that TYLCSV DNAs is hyper-methylated in co-infected plants. Recently, it was established that the heterogeneous linear dsDNA (hdsDNA) forms^49^ are the sole targets of methylation, while supercoiled dsDNA is not methylated^50^. This could suggest that plants co-infected by PSTVd might accumulate more hdsDNA intermediates, likely due to physiological conditions that favor recombination-dependent replication. However, neither recombination-dependent nor rolling circle replication strategies seem compatible with the maintenance of methylation and *de novo* processes that act on linear or circular dsDNA forms^51^. Both these forms are transcriptionally active, a prerequisite for *de novo* methylation driven by viral 24-nt sRNAs, which in TYLCSV mostly derive from the intergenic region^52^, pointing to a TGS-based control of this portion of the genome. However, the even distribution of methylation found along the TYLCSV fragment, encompassing coding and non-coding regions, and the lack of difference between the CG/CHG and the CHH contexts (Fig. 4 and Supplementary Table S3) do not support a transcriptional control of TYLCSV genes mediated by PSTVd. Nonetheless, methylation can also govern resistance to GVs^16^,^53^,^54^ and is occasionally linked to plant recovery^15^,^16^. Since tomato plants do not recover from TYLCSV, likely because of an inefficient methylation of viral DNA, it remains to establish if and how PSTVd subverts mechanisms that protect TYLCSV from methylation.

Several reports have shown that GVs counteract plant PTGS- or TGS-defense through RSSs^15^,^18^ and the modulation of genes mediating methylation pathways^20^,^41^,^55^. In the model here proposed, the viroid generates a condition that alters the plant reaction to GV, directly activating *de novo* DNA methylation and resulting in the hypermethylation of viral replication intermediates. Alternatively, PSTVd could disrupt the interaction between viral and host proteins that regulate the viral methylation or modify the arsenal of anti-viral host defenses. In line with the latter view, the *Ty-1*/*Ty-3* genes conferring tolerance to TYLCV and other GVs encode a RDR and lead to increased methylation of viral DNA^54^. Whether PSTVd affects the expression or the activity of RDRs awaits further investigations.

The major puzzling question arising from our work is how PSTVd, a non-coding RNA, may induce over-expression of methylation-related genes. In this context, it is worth noting that nuclear-replicating viroids lead to over accumulation of salicylic acid (SA) and its metabolic derivative gentisic acid^56^. SA dynamically modulates plant DNA methylation, often coupled to transcriptional changes of transposons and/or proximal genes^57^. Since (i) GVs trigger the SA pathway^46^,^58^, (ii) overexpression of components of the SA pathway impairs GV infectivity^59^,^60^, and (iii) SA interferes with the pathogenicity of the C2 protein of TYLCSV^61^, our findings could result from complex hormonal balances modulated by the dual biotic stresses.

To conclusively prove that PSTVd elicits host DNA methylation, methylome and transcriptome profiles of healthy and infected tomato plants should be compared. Moreover, the use of severe and mild PSTVd strains in co-infection experiments would allow identifying epigenetically-regulated tomato genes that are specifically related to phenotypic alterations.

In summary, our efforts to study plant responses to single and double biotic stresses helped us to dig out the contribution of DNA methylation in the interplay between GVs and viroids in their natural host. This study opens new perspectives about the outcome of multiple biotic stresses in plants. It also revealed the unexpected ability of a nuclear-replicating viroid to stimulate host genes involved in methylation, with clear antagonistic effect on GVs. Dissecting the molecular mechanisms underlying such complex interaction requires further investigations that might disclose novel regulatory networks governing the interplay between the host and these two nuclear-replicating pathogens.

## Materials and Methods

### Plant materials, growth conditions and pathogen inoculation

Tomato (*Solanum lycopersicum*, cv. Marmande) plants were grown in soil, in growth chamber at 25 °C under 16 h light/8 h dark cycle. Plants were inoculated at the 4–5 leaf stage using either *Agrobacterium tumefaciens* LBA4404 carrying the infectious clone of TYLCSV (Genbank Acc. No. X61153)^24^ or *A. tumefaciens* C58 carrying a head-to-tail dimeric cDNA of PSTVd (NB variant; GenBank Acc. No AJ634596.1) under the control of the 35S promoter of *Cauliflower mosaic virus*. For double inoculations, TYLCSV and PSTVd agroclones were mixed at equal volumes before delivery, while for single infections, each agroclone was mixed with *A. tumefaciens* LBA4404 carrying the empty pBIN19 vector. Control plants received only the empty pBIN19. In superinfection experiments, plants received the TYLCSV agroclone alone and, three weeks later, the PSTVd agroclone or the empty pBIN19, as control.

### Symptom and biomass analysis

Plants were scored for symptom development from 2 to 11 weeks post inoculation (wpi). Height and aboveground biomass of individual plants were measured at 11 wpi. Aboveground biomass was determined with an analytical balance following drying at 180 °C for 24 h.

### Virus and viroid detection

For virus detection, DNA was extracted from the third upper leaf from the apex at 6 wpi, using TLES buffer (150 mM LiCl, 50 mM Tris-HCl, pH 9.0, 5 mM EDTA, 5% SDS)^62^. Samples were separated in 1% agarose gels in 0.5X Tris-borate-EDTA (TBE) for 5 h at 80 V; in some instances, ethidium bromide (500 ng/ml) was added to agarose and buffer to separate TYLCSV ss and dsDNA forms. Stained gels were photographed and total genomic DNA loading was recorded. Gels were blotted onto Hybond N^+^ membranes (Roche Diagnostics) and these were hybridized with a digoxigenin-labeled DNA probe specific for the TYLCSV coat protein^63^.

Regarding viroid detection, samples were phenol-extracted^64^ and separated by denaturing PAGE in 5% acrylamide/8M urea gels, in 1X TBE; gels were stained with ethidium bromide to assess equal loading and electrotransferred to Hybond N^+^ membranes (Roche). Membranes were hybridized with a PSTVd-specific digoxigenin-labeled riboprobe at 68 °C in the DIG-Easy Hyb Granules solution (Roche).

### Quantitative RT-PCR for gene expression analysis

RNA samples extracted with Trizol^®^ (Life Technologies) were treated with Turbo DNase free (Ambion). Absence of genomic DNA was evaluated by RT-PCR using the One Step RT-PCR kit (Qiagen) and 18S rRNA-specific primers. For qRT-PCR, cDNA was synthetized from 1.5 μg total RNA with Oligo-dT primers (Invitrogen, Thermo Fischer Scientific) and StrataScript reverse transcriptase (Stratagene). RNA samples were diluted into 40 μl and 10 μl of a mix composed of 0.6 μl of Oligo-dT (500 ng/ml) and 9.4 μl of distilled water were added. Samples were incubated for 5 min at 65 °C and for 10 min at room temperature. A master mix (8.5 μl) containing 5 μl of StrataScript RT buffer, 1 μl of RNase inhibitor (40 U/μl), 2 μl of dNTPs (10 mM), and 0.5 μl of RT StrataScript enzyme was added and samples were incubated at 42 °C for 1 h. Three biological replicates each with three technical repetitions were tested for each virus/gene combination, using the primers listed in Table 1. PCR efficiency was calculated from standard curves obtained using serial dilutions of tomato genomic DNA. The relative expression level of each gene was determined by the Pfaffl method, using UBC (SGN-U582847) as HK gene. The stability of UBC expression in the different infection condition was verified by ANOVA, using all the Mean Threshold Cycle (Ct) values from three plants, with three technical replicates each.

### Methylation dependent restriction analysis

DNA extracted as above from plants infected by TYLCSV or co-infected by TYLCSV/PSTVd (1.5 or 4.2 μg, respectively), harvested at 6 wpi, was restricted with either *Msp*I (cutting only when the inner cytosine is methylated, but blocked if the external cytosine is methylated) or *Hpa*II (cutting only un-methylated DNA) (20 U each, 37 °C for 20 h). A Rolling Circle Amplification (RCA) product obtained using TempliPhi (GE Healthcare) from TYLCSV-infected plants was digested in parallel, as un-methylated DNA control. Undigested (U) DNA from a TYLCSV-infected plant was loaded as further control. As a size marker, a TYLCSV full-length clone made in *Sst*I was digested with either *Sst*I, *Sph*I/*Sna*BI, or *Bgl*II/*Sal*I. Digestion products were combined and 2 ng of the mixture were loaded on gels. All samples were separated in 1% agarose gels in 0.5X TBE (5 h, 80 V). Following ethidium bromide staining, gels were blotted onto nylon membranes, hybridized with a full-length TYLCSV-specific probe labeled with digoxigenin (Roche), according to standard procedures.

### Bisulfite sequencing analysis

Bisulfite conversion was performed on aliquots of 0.8–1 μg DNA prepared as reported above, using the Epiteck Bisulfite kit (Qiagen). As internal control, 50 ng of a purified PCR product corresponding to a cDNA fragment (bp 451–1700, Genbank Acc. No. DQ256073) of the *Pelargonium flower break virus* was added to the reaction mixture. An aliquot (2 ul) of converted samples was amplified using *Taq* DNA polymerase and the Expand High Fidelity PCR system (Roche Applied Science), with the following TYLCSV-specific primers, TY-2428F (nt 2428–2452) ATTGGATGAGAATATGGAGATGAGG and TY-592R (nt 575–592) TTACATCACTAACACAAC, appropriately designed to minimize cytosine content. For TYLCSV, the PCR protocol encompassed 35 cycles, each consisting of denaturation at 94 °C for 30 s, primer annealing at 52° C for 30 s, extension at 72 °C for 60 s. For the bisulfite control, primer annealing was at 50 °C and extension at 72° C for 30 s, for 30 cycles. In both cases, the reaction ended with a final elongation step of 7 min at 72 °C. PCR products were electrophoresed on 1.2% agarose gels in 1X TAE (0.04 M Tris-acetate, 1 mM EDTA). Following ethidium bromide staining, amplicons were recovered from gel using Quantum Prep^TM^ Freeze ′N Squeeze DNA Gel Extraction Spin Columns (Bio-Rad), cloned into pGEM-T Easy Vector (Promega), transformed into competent *Escherichia coli* DH5α cells and custom-sequenced (MWG Biotech AG). Sequences from converted and unconverted DNA were aligned to compare methylated and un-methylated cytosines in the different contexts.

## Additional Information

**How to cite this article**: Torchetti, E. M. *et al*. A nuclear-replicating viroid antagonizes infectivity and accumulation of a geminivirus by upregulating methylation-related genes and inducing hypermethylation of viral DNA. *Sci. Rep*. **6**, 35101; doi: 10.1038/srep35101 (2016).

## Supplementary Material

Supplementary Information

## Figures and Tables

**Table 1:** List of primers used for quantitative RT-PCR.

Tomato gene	Gene acronym	Gene identification	Primer name	Primer sequence 5′ > 3′
S-adenosyl-l-homocysteine hydrolase	SAHH	AK322109	807F	CACTCACTTCCCGATGGTCT
			929R	GCACCAGCTTGTTTCATGG
Adenosine kinase	ADK	BT012983	606F	ACGCAGCAGCCAATAACAA
			737R	TCTTGCTTCTGTCTCATTTCCA
S-adenosyl-l-methionine synthetase	SAMS	BT012699	632F	CTGATGGCAAGACCCAAGTT
			740R	TAACGGTCTCATCGTGTTGG
Hua Enhancer 1	HEN1	SGN-U575788	367F	GCTAGCTCAGATGCCCATTC
			477R	GGAGGATGACCCAGATGAGA
Chromomethylase 3	CMT3	SGN-U582753	703F	GTCAAATTCCAAAGCGGAAG
			852R	CGAAGCTCATCGCATAGTCA
Deficient in DNA Methylation 1	DDM1	SGN-U603273	58F	ACAGGCTATGGATCGGTGTC
			195R	CCAATCACCACATGCTCAAG
Domains rearranged methyltransferase 2	DRM2	SGN-U577457	1444F	ATTGGGGTTTCCAAAGAACC
			1612R	GCCAATCCCAGAGAAGAGTG
Methyltransferase 1	MET1	SGN-U569088	2594F	ACTCACCCGAGGTGTCAAAG
			2696R	TTCCTCTCCGGAACATCATC
S-adenosyl-methionine decarboxylase 1	SAMDC1	SGN-U580064	762F	TATGTGCTGTCCGAGTCGAG
			876R	AGGTCTCAGCCAACCTCAGA
Argonaute 4a	AGO4a	JX467709	2696F	GCTACTCAAATGGGACAGTGG
			2869R	AAGAAAAGCATGAAGGCGTTAC
Nuclear RNA Polymerase D1A	NRPD1	SGN-U602663	376F	GGGGATTTTGATGGTGATTG
			485R	TTCTGTCCGTCAAGCAACTG
Nuclear RNA Polymerase E1	NRPE1	SGN-U596095	496F	CAGTTCGAATATGGGTCTAGCG
			620R	GATGAATCCAGAACTGCCTTGT
Repressor Of Silencing 1	ROS1	SGN-U575521	301F	CAAATGTTGGGCGTATAGCTG
			409R	TGAATTGACTCCAGCACAGG
Ubiquitin Conjugating Enzyme	UBC	SGN-U582847	418 F	ACCTGATGATCCACAAGATGC
			535 R	GCAGCAAGTGTGGATGTTTTT
