# Supplementary material for: A nuclear-replicating viroid antagonizes infectivity and accumulation of a geminivirus by upregulating methylation-related genes and inducing hypermethylation of viral DNA
Source: Sci Rep. 2016 Oct 14;6:35101. doi: 10.1038/srep35101 (PMC5064398; doi:10.1038/srep35101)
Supplement: Supplementary Information [file srep35101-s1.pdf]

SUPPLEMENTARY INFORMATION FOR

A nuclear-replicating viroid antagonizes infectivity and accumulation of a geminivirus by upregulating methylation-related genes and inducing hypermethylation of viral DNA

EM Torchetti, M Pegoraro, B Navarro, M Catoni, F Di Serio, E Noris

Supplementary Table S1. Expression of the housekeeping gene Ubiquiting Conjugating enzyme in the different experimental conditions.

Mean Threshold Cycles (Ct)				Significativity
Non Infected	TYLCSV	PSTVd	TYLCSV/ PSTVd	
24.926	26.132	23.633	24.004	
25.083	26.574	25.191	23.984	0.076
25.050	24.541	24.295	24.263	

Analysis of variance (ANOVA) of the Mean Ct values obtained from three biological replicates per treatment, each with three technical replicates.

Supplementary Table S2. P-values of the fold changes of gene expression calculated for each experimental conditions.

Tomato gene	TYLCSV vs. mixed infection	TYLCSV vs. PSTVd	PSTVd vs. mixed infection
MET1	0.010	0.024	0.446
CMT3	0.006	0.019	0.488
DDM1	0.009	0.023	0.500
KYP	0.025	0.050	0.527
IBM1	0.044	0.063	0.476
DRM2	0.020	0.050	0.896
NRPD1	0.018	0.073	0.816
NRPE1	0.018	0.037	0.367
HEN1	0.018	0.048	0.211
AGO4a	0.019	0.038	0.767
ADK	0.026	0.060	0.633
SAHH	0.027	0.033	0.711
SAMS	0.035	0.050	0.581
SAMDC1	0.008	0.009	0.879
ROS1	0.015	0.021	0.910

Supplementary Table S3. P-values of the percentage of methylated cytosines in the TYLCSV region analyzed, calculated for the total cytosines and for each cytosine context.

Cytosine context	TYLCSV vs. mixed infection
**Total**	1.92 E-08
**CHH**	2.13 E-09
**CHG**	1.81 E-07
**CG**	2.42 E-06

A

CCAACGAGCTGGCCGTAATGAAGTATCTCGTGGGTAAGTGCCGCGAGCACCGCTTAGTGGTGCAGCACACCCGCGAAGTCTGCTCTCTGGCGATGGCTGCTATTTTCACCCCAGACCACCATGAA GTAAACATGGT
TAGGGAGATGAACAGCCACGCTGCCTACCGAAGAAGGGTAGCTCTTGCAGATGCCGCCAGAGTCCATAGCTGGCAGCAGGAGCTACTCCGCTCACCCCTGAGTTGGAACGC TTGGGGGAGGGCGTGGTGGATAGCC
AACGGGCTTCCTGACAGGGAGCCTGTCCGGTTCACCAAATAGGGGGGTCTTTTCTGCCTCGAGGGTGTTGAAACAGGAGTCCGTAGGGGGACGCACCCACAGATGATCGAGGTAGAGAAAAACTC CCCCCCGAAAC
TTAGGAAACTCTTCGCCCAACGGTTGGTTAGCAGTGGAGTTGAATACCGCGTGCATAACCACTCGTTTCAAAACCTAAGGCGAGGTGTGCTTGAGAGAGTTTTCTACGTGGAAGAAAATAAGCAG TTAGTATCGTG
TCCCTGTCCGGAAAAGGGGGCCTTCAAGGAAATGGGTTACCTACGAAGGCAATTCGTTAGACTTACTCCCAAACATGCCAGGATCTCAGCAAAGGAATTTGTTGATTGCTATC ATGGTAGGAAGAAGAGTGTCTAC
GAGTTTGCTGCCATGACCCTCTCTGAGAGACCTCTGGACCGCAAGGATGCCAGCTTGAAGACGTTTATAAAGGCCGAAAAATTTTGCAAGGCCGACCCAGCTCCCCGGGTTATACAACCAAGAAG TCCGCGCTACA
ACGTTGAATTAGGCCGATATCTCAAGAAATTCGAACACTCGGCCTACAGAACGCTGGACAAGATCTGGGGAGGAAAGACCGTGATGAAAGGGTACTCTGTGGATGAGATAGGGAAACATATCTCA GAGGCGTGGGA
TTCATTCCACTCTCCGGTTGCTGTCGGATTCGACATGAGCCGGTTCGACCAGCACGTTAGTGTGCCTGCGCTTCAATTCGAACACAGTTGTTACCTTGCCTGTTTTCCCGGAGACCAGCATTTGG CGGAACTCTTG
AGCTGGCAGCTCAAGAATTTTGGCGTAGGGTTCGCGTCAAATGGAATGGTTAGATATAAGAAGGATGGGTGTAGAATGTCCGGAGACATGAACACTGCACTGGGAAACTGCCTATTGGCTTGCCT CATAACTAGGC
ACCTCATGAAGGACATAAATTGTCGA

B

	721	722	725	729	732	733	737	744	745	749	750	755	757	758	771	776	779	780	782	796	803	804	814	816	818	819	820	822	830	845	847	848	849	850	851	852	853	858	867	869	872	874	875	876	879
Control	C	C	C	C	C	C	C	C	C	C	C	C	C	C	C	C	C	C	C	C	C	C	C	C	C	C	C	C	C	C	C	C	C	C	C	C	C	C	C	C	C	C	C	C	C
TY_1C1	T	T	T	T	T	T	T	T	T	T	T	T	T	T	T	T	C	T	T	T	T	T	T	T	T	T	T	T	T	T	T	T	T	T	T	T	T	T	T	T	T	T	T	T	T
TY_1C2	T	T	T	T	T	T	T	T	T	T	T	T	T	T	T	T	T	T	T	T	T	T	T	T	T	T	T	T	T	T	T	T	T	T	T	T	T	T	T	T	T	T	T	T	T
TY_1C3	T	T	T	T	T	T	T	T	T	T	T	T	T	T	T	T	T	T	T	T	T	T	T	T	T	T	T	C	T	T	T	T	T	T	T	T	T	T	T	T	T	T	T	T	T
TY_1C4	T	T	T	T	T	T	T	T	T	T	T	T	T	T	T	T	T	T	T	T	T	T	T	T	T	T	T	T	T	T	T	T	T	T	T	T	T	T	T	T	T	T	T	T	T
TY_2C1	T	T	T	T	T	T	T	T	T	T	T	T	T	T	T	T	T	T	T	T	T	T	T	T	T	T	T	T	T	T	T	T	T	T	T	T	T	T	T	T	T	T	T	T	T
TY_2C2	T	T	T	T	T	T	T	T	T	T	T	T	T	T	T	T	T	T	T	T	T	T	T	T	T	T	T	T	T	T	T	T	T	T	T	T	T	T	T	T	T	T	T	T	T
TY-2C3	T	T	T	T	T	T	T	T	T	T	T	T	T	T	T	T	T	T	T	T	T	T	T	T	T	T	T	T	T	T	T	T	T	T	T	T	T	T	T	T	T	T	T	T	T

	890	905	906	908	912	917	918	920	922	927	932	933	939	948	962	965	981	991	996	997	998	1002	1003	1015	1016	1019	1034	1035	1038	1044	1049	1056	1060	1063	1063	1064	1068	1072	1073	1079	1081	1084	1103	1107
Control	C	C	C	C	C	C	C	C	C	C	C	C	C	C	C	C	C	C	C	C	C	C	C	C	C	C	C	C	C	C	C	C	C	C	C	C	C	C	C	C	C	C	C	C
TY_1C1	T	T	T	T	T	T	T	T	T	T	T	T	T	T	T	T	T	T	T	T	T	T	T	T	T	T	T	T	T	T	T	T	T	T	T	T	T	T	T	T	T	T	T	T
TY_1C2	T	T	T	T	T	T	T	T	T	T	T	T	T	T	T	T	T	T	T	T	T	T	T	T	T	T	T	T	T	T	T	T	T	T	T	T	C	T	T	T	T	T	T	T
TY_1C3	T	T	T	T	T	T	T	T	T	T	T	T	T	T	T	T	T	T	T	T	T	T	T	T	T	T	T	T	T	T	T	T	T	T	T	T	T	T	T	T	T	T	T	T
TY_1C4	T	T	T	T	T	T	T	T	T	T	T	T	T	T	T	T	T	T	T	T	T	T	T	T	T	T	T	T	T	T	T	T	T	T	T	T	T	T	T	T	T	T	T	T
TY_2C1	T	T	T	T	T	T	T	T	T	T	T	T	T	T	T	T	T	T	T	T	T	T	T	T	T	T	T	T	T	T	T	T	T	T	T	T	T	T	T	T	T	T	T	T
TY_2C2	T	T	T	T	T	T	T	T	T	T	T	T	T	T	T	T	T	T	T	C	T	T	T	T	T	T	T	T	T	T	T	T	T	T	T	T	T	T	T	T	T	T	T	T
TY-2C3	T	T	T	T	T	T	T	T	T	T	T	T	T	T	T	T	T	T	T	T	T	T	T	T	T	T	T	T	T	T	T	T	T	T	T	T	T	T	T	T	T	T	T	T

C

	721	722	725	729	732	733	737	744	745	749	750	755	757	758	771	776	779	780	782	796	803	804	814	816	818	819	820	822	830	845	847	848	849	850	851	852	853	858	867	869	872	874	875	876	879
Control	C	C	C	C	C	C	C	C	C	C	C	C	C	C	C	C	C	C	C	C	C	C	C	C	C	C	C	C	C	C	C	C	C	C	C	C	C	C	C	C	C	C	C	C	C
TYPS_1C1	T	T	T	T	T	T	T	T	T	T	T	T	T	T	T	T	T	T	T	T	T	T	T	T	T	T	T	T	T	T	T	T	T	T	T	T	T	T	T	T	T	T	T	T	T
TYPS_1C2	T	T	T	T	T	T	T	T	T	T	T	T	T	T	T	T	T	T	T	T	T	T	T	T	T	T	T	T	T	T	T	T	T	T	T	T	T	T	T	T	T	T	T	T	T
TYPS_1C3	T	T	T	T	T	T	T	T	T	T	T	T	T	T	T	T	T	T	T	T	T	T	T	T	T	T	T	T	T	T	T	T	T	T	T	T	T	T	T	T	T	T	T	T	T
TYPS_1C4	T	T	T	T	T	T	T	T	T	T	T	T	T	T	T	T	T	T	T	T	T	T	T	T	T	T	T	T	T	T	T	T	T	T	T	T	T	T	T	T	T	T	T	T	T
TYPS_2C1	T	T	T	T	T	T	T	T	T	T	T	T	T	T	T	T	T	T	T	T	T	T	T	T	T	T	T	T	T	T	T	T	T	T	T	T	T	T	T	T	T	T	T	T	T
TYPS_2C2	T	T	T	T	T	T	T	T	T	T	T	T	T	T	T	T	T	T	T	T	T	T	T	T	T	T	T	T	T	T	T	T	T	T	T	T	T	T	T	T	T	T	T	T	T
TYPS_2C3	T	T	T	T	T	T	T	T	T	T	T	T	T	T	T	T	T	T	T	T	T	T	T	T	T	T	T	T	T	T	T	T	T	T	T	T	T	T	T	T	T	T	T	T	T

	890	905	906	908	912	917	918	920	922	927	932	933	939	948	962	965	981	991	996	997	998	1002	1003	1015	1016	1019	1034	1035	1038	1044	1049	1056	1060	1063	1063	1064	1068	1072	1073	1079	1081	1084	1103	1107
Control	C	C	C	C	C	C	C	C	C	C	C	C	C	C	C	C	C	C	C	C	C	C	C	C	C	C	C	C	C	C	C	C	C	C	C	C	C	C	C	C	C	C	C	C
TYPS_1C1	T	T	T	T	T	T	T	T	T	T	T	T	T	C	T	T	T	T	T	T	C	T	T	T	T	T	T	T	T	T	T	T	T	T	T	T	T	T	T	T	T	T	T	T
TYPS_1C2	T	T	T	T	T	T	T	T	T	T	T	T	T	T	T	T	T	T	T	T	T	T	T	T	T	T	C	T	T	T	T	T	T	T	T	T	T	T	T	T	T	T	T	T
TYPS_1C3	T	T	T	T	T	T	T	T	T	T	T	T	T	T	T	T	T	T	T	T	T	T	T	T	T	T	T	T	T	T	T	T	T	T	T	T	T	T	T	T	T	T	T	T
TYPS_1C4	T	T	T	T	T	T	T	T	T	T	T	T	T	T	T	T	T	T	T	T	T	T	T	T	T	T	[illegible]	T	T	T	T	T	T	T	T	T	T	T	T	T	T	T	T	T
TYPS_2C1	T	T	T	T	T	T	T	T	T	T	T	T	T	T	T	T	T	T	T	T	T	T	T	T	T	T	T	T	T	T	T	T	T	T	T	T	T	T	T	T	T	T	T	T
TYPS_2C2	T	T	T	T	T	T	T	T	T	T	T	T	T	T	T	T	T	T	T	T	T	T	T	T	T	T	T	T	T	T	T	T	T	T	T	T	T	T	T	T	T	T	T	T
TYPS_2C3	T	T	T	T	T	T	T	T	T	C	T	T	T	T	T	T	T	T	T	T	T	T	T	T	T	T	T	T	T	T	T	T	T	T	T	T	T	T	T	T	T	T	T	T

Supplementary Figure S1. Methylation of the internal DNA control used in bisulfite conversion experiments. (**A**) cDNA fragment of *Pelargonium flower break virus* genomic RNA (Genbank Acc. No. DQ256073; nt 451-1700), added (50 ng) to the bisulfite reactions performed to establish TYLCSV DNA methylation in TYLCSV- and TYLCSV/PSTVd-infected tomato plants (see Figure 4). A bisulfite-converted DNA fragment (387 bp, nt 721-1108) was amplified using forward and reverse primers (underlined in **A**), cloned and sequenced. **B**,**C.** Individual clones of the bisulfite-converted controls of the results reported for TYLCSV DNA in Fig. 4**B**,**C**, respectively. Methylated cytosines (indicated with C) are grey, red or green, if in the CHH, CHG and CG contexts, respectively. Unmethylated cytosines are reported as T. Conversion efficiency was > 99% in each replicate.

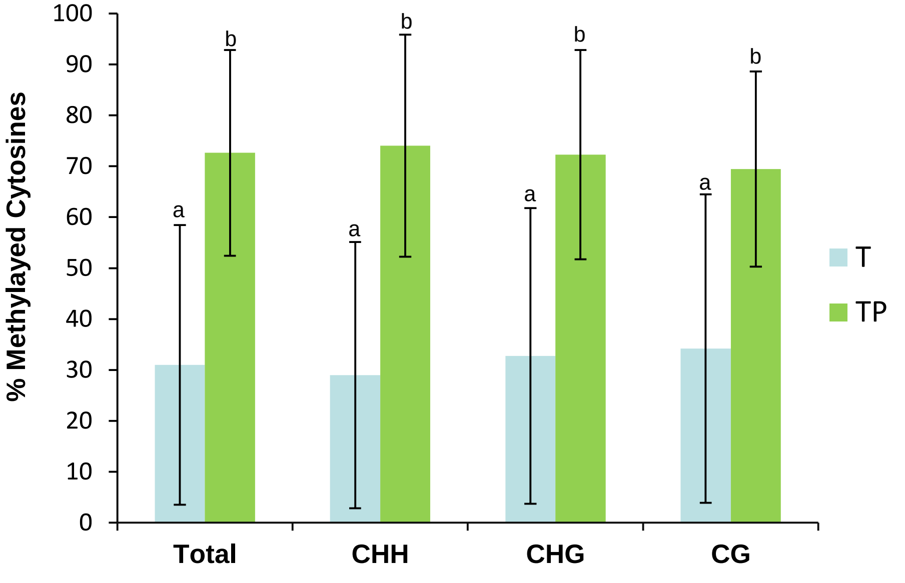

Supplementary Figure S2. Bisulfite sequencing analysis of TYLCSV genome methylation in tomato plants infected by TYLCSV alone (T) or doubly infected by TYLCSV and PSTVd (TP). Columns represent mean percentage of methylated cytosines in the TYLCSV region analyzed independently from the DNA context (Total) or taking into consideration the CHH, CHG and CG contexts. Bars, standard deviation; letters, statistical significance (P≤0.0001; Student t-test).
